# A Machine-Learning-Based Approach for Predicting Mechanical Performance of Semi-Porous Hip Stems

**DOI:** 10.3390/jfb14030156

**Published:** 2023-03-15

**Authors:** Khaled Akkad, Hassan Mehboob, Rakan Alyamani, Faris Tarlochan

**Affiliations:** 1 Department of Engineering Management, Prince Sultan University, P.O. Box 66833, Riyadh 11586, Saudi Arabia; 2 Department for Mechanical and Industrial Engineering, Qatar University, Doha 2713, Qatar

**Keywords:** machine learning, hip stem, finite element analysis

## Abstract

Novel designs of porous and semi-porous hip stems attempt to alleviate complications such as aseptic loosening, stress shielding, and eventual implant failure. Various designs of hip stems are modeled to simulate biomechanical performance using finite element analysis; however, these models are computationally expensive. Therefore, the machine learning approach is incorporated with simulated data to predict the new biomechanical performance of new designs of hip stems. Six types of algorithms based on machine learning were employed to validate the simulated results of finite element analysis. Afterwards, new designs of semi-porous stems with outer dense layers of 2.5 and 3 mm and porosities of 10–80% were used to predict the stiffness of the stems, stresses in outer dense layers, stresses in porous sections, and factor of safety under physiological loads using machine learning algorithms. It was determined that decision tree regression is the top-performing machine learning algorithm as per the used simulation data in terms of the validation mean absolute percentage error which equals 19.62%. It was also found that ridge regression produces the most consistent test set trend as compared with the original simulated finite element analysis results despite relying on a relatively small data set. These predicted results employing trained algorithms provided the understanding that changing the design parameters of semi-porous stems affects the biomechanical performance without carrying out finite element analysis.

## 1. Introduction

Conventional hip stems are made of dense metals such as cobalt-chromium, stainless steel, and titanium. The stiffness of these dense stems is 5–15 fold higher than that of the cortical bone and 50–100 fold higher than that of the cancellous bone [1,2]. This high mismatch of stiffness causes several complications such as stress shielding, aseptic loosening, corrosion, and implant failure [3,4]. Thus, revision of surgeries is required to fix the failed stems, which is a costly and painful process. To overcome the aforementioned complications, researchers have developed fascinating materials and designs using the additive manufacturing technique to reduce the stiffness of stems that can last longer with excellent functionality [5,6]. The choices of materials are limited due to biocompatibility; however, the implant designs have been extensively studied in recent decades. Several studies have shown excellent biocompatibility of titanium alloys when tested in vivo. Titanium alloys are inert and showed excellent bone ingrowth into the porous surfaces [7,8,9]. Moreover, advanced manufacturing techniques such as additive-manufactured implants also showed excellent biocompatibility; however, the development of new materials and designs of medical devices does not contain biocompatibility testing until the prototype stage is reached [8]. Recently, different stem designs were based on the additive manufacturing concept that provides wide freedom to alter the design [10,11,12]. Porous stems with various architectures are manufactured with additive manufacturing to reduce the stiffness of the stems. These porous cellular architectures include circular, cubic, body-centered cubic, diamond, and gyroid shapes [13]. Moreover, the porosity of these cells can be easily controlled within stems. These porous structures are usually filled inside the stem with an outer dense layer; thus, these design parameters generate a lot of design alternatives that are costly and time-consuming, using experiments. Therefore, computer models [14,15] are attracting the attention of researchers to reduce the cost, time, and uncertainty in experimental work. Alkhatib et al. [14] investigated the biomechanical performance of hip stems under various physiological conditions such as walking and stair climbing, and found that hip stems showed more stresses in the climbing activity as compared with the walking activity. Moreover, porous hip stems alleviated stress shielding in surrounding bone as compared with dense stems under different activities. However, these simulations are also costly, as they require the running of hundreds of simulations for these design parameters. Thus, researchers have recently given more attention to incorporating machine learning (ML) techniques in the medical field, which are capable of predicting the results [16,17,18].

Machine learning extends to a large variety of fields and can assume different approaches. However, one simple and clear definition was presented by Murphy [19] stating that machine learning is “a set of methods that can automatically detect patterns in data, and then use the uncovered patterns to predict future data”. In other words, machine learning techniques are statistical models that can be autonomously trained to predict data using complex algorithms [20]. Since the last decade, the technique has grown tremendously and is now being used in many fields such as industrial [21], project management [22], finance [23,24], construction and materials [25,26], and medicine [27], among others, to predict multiple types of data. It is worth mentioning that machine learning methods can be divided into classifiers and predictors. Classifiers are used for categorical variables whereas predictors are used for numerical variables, the latter being the case in this paper.

In medicine, several researchers have turned to ML techniques as a computationally cheaper alternative to Finite Element (FE) model simulations. Villamor et al. [28] made comparisons between Support Vector Machine (SVM), Logistic Regression, Shallow Neural Networks, and Random Forest ML methods to determine the best-performing model to predict osteoporotic hip fracture in postmenopausal women based on FE analyses. Alastruey-López et al. [29] used Artificial Neural Networks (ANN) and a parametric FE simulation to predict impingement and dislocation in total hip arthroplasty. Their efforts were aimed at identifying the optimal prosthesis design to reduce the probability of dislocation. Similarly, Jun et al. [30] used results produced using an FE model analysis to train a machine learning method that combines both principal component analysis (PCA) and support vector regression (SVR) in an effort to predict the contact stress of the hip prosthesis acetabular lining. The prediction model performance was then compared with the ridge regression and lasso models for validation. Cilla et al. [20] also combined FE modeling, ANNs, and SVMs in an effort to optimize the commercial short-stem hip prosthesis design. Their work focused on predicting the optimal stem length, thickness in the lateral and medial, and the distance between the implant neck and the central stem surface.

Within this context, this study aims to address stress shielding in dense hip implants by introducing a porous hip implant. ML techniques were used to validate finite element results of porous hip stems with different designs. Six types of algorithms were used to investigate errors. Then, a new data set was created with semi-porous hip stems which have outer dense layers with different thicknesses and inner porous cellular structures with different porosities. The most efficient validated algorithm was used to predict the outcomes of new implant designs, which are stiffness of the implants, stresses in dense layers and porous cellular structures, and factor of safety of the implants. 

There are multiple stem designs that use finite element analysis (FEA) to investigate biomechanical performance, as mentioned above. One of these unique designs that help alleviate stress shielding was proposed by Mehboob et al. [12]. Accordingly, this paper contributes to the existing literature as follows:

1. Using predictive machine learning techniques to validate the FEA-based models presented by Mehboob et al. [12] to reduce the in vivo experimental cost.
2. Comparing multiple machine learning algorithms to determine the best-performing method for the chosen model.

Quick prediction of the designs of the stems using machine learning will be readily available for printing as a personalized implant which will decrease the complications of revision of surgery and reduce the burden on the health system. In addition, the lower cost achieved through machine-learning-aided designs will potentially reduce the cost of the implant on patients. Moreover, designing the semi-porous implants and printing using additive manufacturing will further reduce the wastage of the material. 

The remainder of this paper is organized as follows. Section 2 explains the methodology used in the development of the hip implant through a finite element analysis and the training data set, as well as the methodology for developing the predictive models using machine learning techniques. Section 3 presents and discusses the results obtained from all six machine learning models in addition to identifying the best-performing model as well as an interpretation of these results. The final section presents an overall conclusion in addition to some limitations and avenues for future research.

## 2. Methodology

### 2.1. Finite Element Analysis

**Figure 1:**
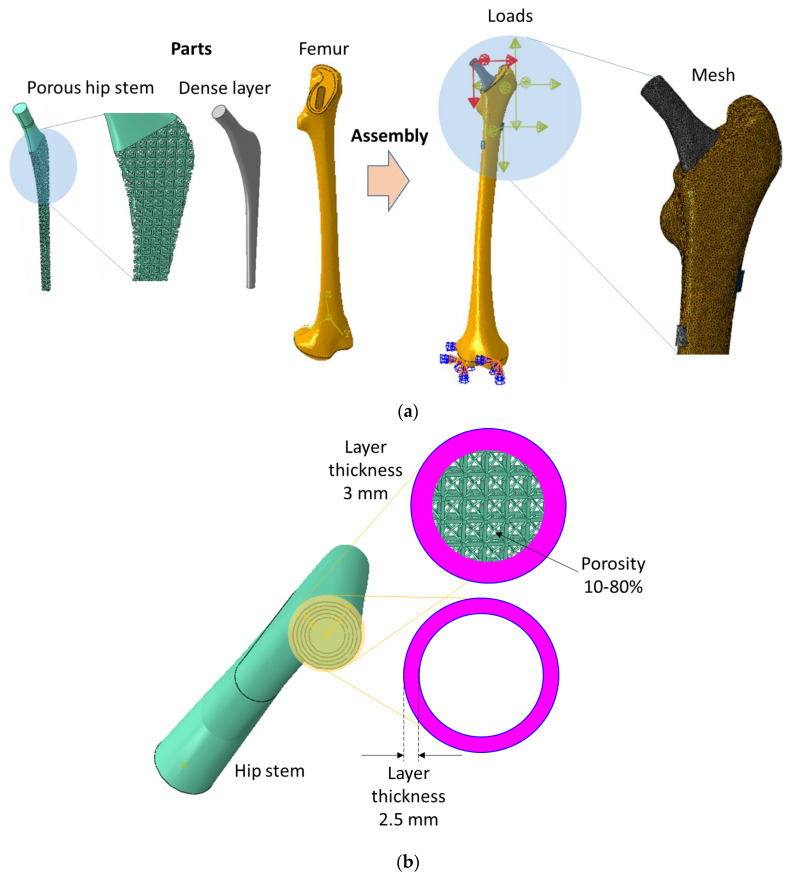
Finite element models of semi–porous stems; (**a**) finite element models used in the previous study [12], (**b**) new design parameters for prediction of biomechanical performance using machine learning.

Finite element models of various designs of hip implants and bone were constructed in SolidWorks in a previous study [12]. These models were assembled in simulation code, ABAQUS v6.17 (Dassault Systemes, Vélizy-Villacoublay, France), and physiological loads and boundary conditions were applied to mimic a realistic situation, as shown in Figure 1a. A parametric study of the influence of hip implant design on the stiffness of stems, stresses in implants, and fatigue life in terms of the factor of safety was investigated in a previous study [12]. In the previous study [12], the layer thickness and porosity were changed to investigate the factor of safety. The factor of safety was calculated using the Soderberg approach ($\frac{\sigma_{a}}{S_{e}}\;$+$\;\frac{\sigma_{m}}{S_{y}}=\frac{1}{n}$), where, σ_a_ is stress amplitude, σ_s_ is mean stress, S_e_ is endurance limit, S_y_ is the yield strength of the material and *n* is the factor of safety. The factor of safety is calculated to determine the safety of the structure under a certain load [31]. For instance, if the factor of safety is greater than 1 for a certain load, then the structure is considered safe under that load; otherwise, the structure is considered unsafe. In the Soderberg approach, the material properties and the results of simulations were used to calculate the factor of safety. However, these finite element simulations and post-processing calculations are time-consuming and computationally expansive compared with machine learning approaches. In this study, values of inputs of stem designs (layer thickness and porosity) and results (stresses, stiffness, and factor of safety), obtained from Mehboob et al. [12], were used to train the machine learning algorithms and a new test dataset was created based on layer thickness and porosities to predict the stiffness of stems, stresses in implants, and the factor of safety based on previously trained values.

In this study, the data was used to feed and train six types of algorithms. These algorithms were validated with the published data and errors were calculated. After training these algorithms, new design parameters of the hip stem were created with different thicknesses of outer dense layers (2.5 and 3 mm) and inner porosities (10–80%) as shown in Figure 1b. The validated algorithms were used to predict the stiffness of stems, stresses in outer dense layers and porous sections, and factor of safety of newly designed implants. 

### 2.2. Machine Learning

The dataset used in this research is based on finite element analysis as mentioned in Section 2.1. Machine learning algorithms are used to predict the outputs of the simulations for various design parameter settings. The input variables are dense layer thickness (DLT) in mm and porosity of porous section (PPS) in percent: *v*_1_ and *v*_2_, respectively. The output variables, *v*_3_ to *v*_6_, are stiffness of stems (SS) in N/mm, maximum stresses in dense layer (MSDL) in MPa, maximum stresses in porous section (MSPS) in MPa, and factor of safety (FS). Table 1 shows the dataset of dimension 30 × 6. 

The dataset requires minor preprocessing before machine learning algorithms are utilized for the purposes of predicting the simulated results. A MinMax normalization was applied to obtain a normalized dataset in the range [0.1, 1]. It is noted that a normalization of range [0, 1] is problematic due to the upcoming calculations of prediction accuracies. The MinMax normalization is applied using the following equation.

(1) $$normalized\;input=\frac{v_{ij}-\min\left(v_{ij}\right)}{\max\left(v_{ij}\right)-\min\left(v_{ij}\right)}$$

where *i* is the input variable number and *j* is the simulation record. No data imputation techniques were used due to the lack of missing values in the dataset. The entire dataset is then vertically split into *x* = [*v*_1_, *v*_2_] and *y* = [*v*_3_, *v*_4_, *v*_5_, *v*_6_]. A shuffle split is then horizontally implemented to divide the dataset into training which accounts for 70% and validation which accounts for the remaining 30% of the dataset. The resulting training and validation matrices are of dimensions 21 × 6 and 9 × 6, respectively. The vertical split is shuffled using a NumPy seed [32] to allow for a non-sequential sampling with replacement which is next implemented 10 times for each of the utilized machine learning algorithms.

As mentioned earlier, machine learning techniques are generally categorized into classification and prediction algorithms. The machine learning algorithms used in this paper are generally prediction based as the output to be predicted is numerical. Six different algorithms are used, namely decision tree regression (DTR), linear regression, ridge regression (RR), lasso regression (LSR), elastic net (EN), and multilayer perceptron (MLP) regression. Each of the previously mentioned techniques is implemented using its default hyperparameter settings, as shown in Table 2, as per the Scikit-learn library [33]. A brief about each of the machine learning algorithms is presented next. 

#### 2.2.1. Decision Tree Regression (DTR) 

**Figure 2:**
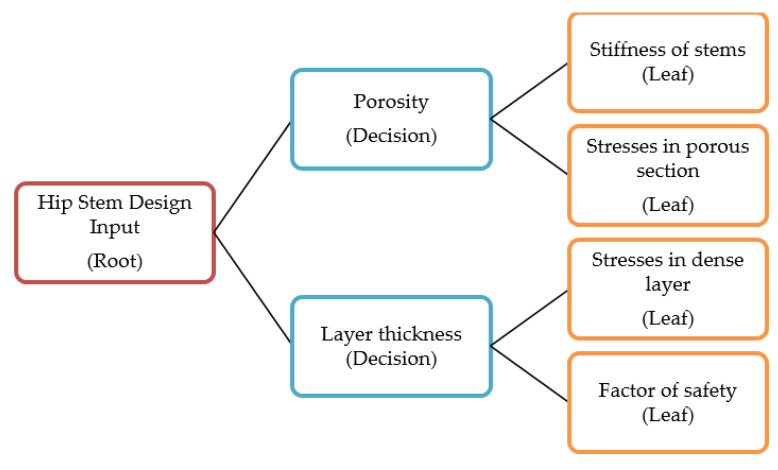
Decision tree structure showing root, decision, and leaf nodes as it relates to the implemented model.

Decision trees can be used for both classification and regression tasks [34]. More specifically, decision trees can be referred to as classification trees and regression trees depending on the studied task. A decision tree regressor or a regression tree is used in this paper in order to predict numerical values. A decision tree is considered to be a collection of splits based on threshold values at the training set level. The information obtained from a trained decision tree is then applied to validation and test sets for the purposes of prediction. A decision tree contains root nodes, decision nodes for splitting, and leaf nodes where the final results are shown. A simple demonstration of the decision tree structure, inspired by Bulbul et al. [34], is presented in Figure 2. In Figure 2, decision nodes are where specific variable values are decided upon, to be assigned to one of the following two leaves based on a criterion established in the decision node. This criterion can be a threshold value for numerical variables or a voting system in categorical variables. Variables are ordered based on which variable is a better splitter of the data to produce more accurate predictions. It should be noted that decision nodes in Figure 2 are simultaneously used for the prediction of all four leaf nodes but can be subdivided as depicted in the figure. 

#### 2.2.2. Linear Regression (LR)

One of the most commonly used machine learning predictors is linear regression. It has been shown to aid in the prediction of output variables based on input variables by means of best-fit linear relationship navigation [35] which is done using least squares minimization. The following equation illustrates the mechanism of the linear regression predictor.

(2) $$Y=\beta_{0}+\sum_{n=1}^{N}\beta_{n}X_{n}+\varepsilon_{n}$$

where Y is the output vector, $X_{n}$ are the multiple input variables, $\beta_{0}$ is a constant, $\beta_{n}$ is the estimated linear parameter signified by the slope which is the regression coefficient, and $\varepsilon_{n}$ is the error. According to Ogutu et al. [36], and for the purposes of illustration, a basic linear regression model can be written as in the following equation.

(3) $$y=µ1_{n}+X\beta +e$$

where β is a vector of coefficients and e is the residual error vector.

#### 2.2.3. Ridge Regression (RR)

Ridge regression is an extension of linear regression [36] where $\ell_{2}$ regularization is used as shown in the following equation.

(4) $$\hat{\beta }\left(ridge\right)=\underset{\beta }{\arg\min}\|y-X\beta {\|}_{2}^{2}+\lambda \|\beta {\|}_{2}^{2}\;$$

where $\|y-X\beta {\|}_{2}^{2}=\overset{n}{\underset{i=1}{\sum }}{\left(y_{i}x_{i}^{T}\beta \right)}^{2}$ is the loss function with $\ell_{2}$ regularization. 

#### 2.2.4. Lasso Regression (LSR)

Another extension of linear regression is lasso regression [36] where $\ell_{1}$ regularization is utilized as presented next.

(5) $$\hat{\beta }\left(lasso\right)=\underset{\beta }{\arg\min}\|y-X\beta {\|}_{2}^{2}+\lambda \|\beta {\|}_{1}$$

where $\|\beta {\|}_{1}=\overset{p}{\underset{j=1}{\sum }}\left|\beta_{j}\right|$ is the loss function with $\ell_{1}$ regularization.

#### 2.2.5. Elastic Nets (EN)

The third and final extension of linear regression that is utilized in this paper is elastic nets. It utilizes both $\ell_{1}$ and $\ell_{2}$ regularization [36]. It is useful for high-dimensional data, which is not the case in this paper. Nonetheless, EN was applied for the purposes of comparison. Elastic nets can be mathematically described as shown in the following equation.

(6) $$\hat{\beta }\left(EN\right)=\left(1+\frac{\lambda_{2}}{n}\right)\left\{\underset{\beta }{\arg\min}\|y-X\beta {\|}_{2}^{2}+\lambda_{2}\|\beta {\|}_{2}^{2}+\lambda_{1}\|\beta {\|}_{1}\right\}$$

#### 2.2.6. Multilayer Perceptron (MLP)

**Figure 3:**
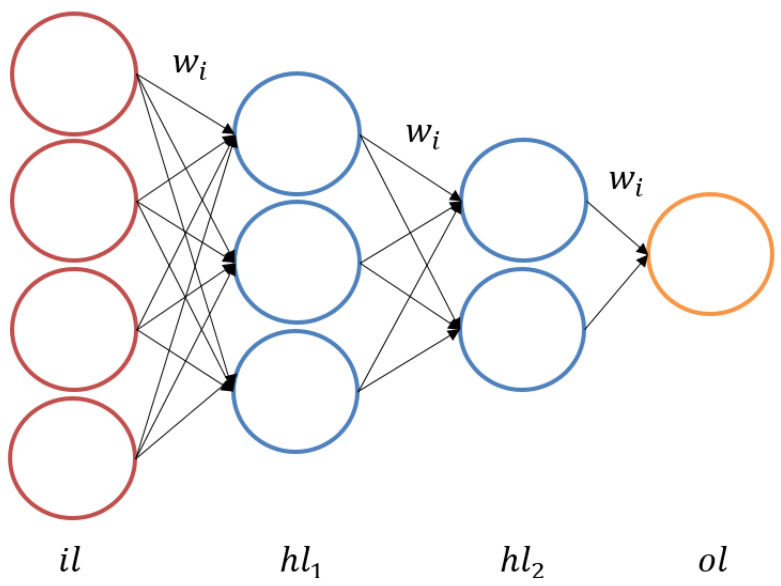
MLP basic structure shows an input layer, two hidden layers, and an output layer.

An artificial neural network, also called a multilayer perceptron, can be utilized in its most basic form for the purposes of supervised learning. It nonlinearly maps inputs to outputs by utilizing nodes and their associated weights [37]. The weights connect the nodes to produce the output which is considered to be the sum of inputs. This mapping is implemented using an activation function which is the rectified linear unit (Relu) in this paper. More specifically, weights are updated through a backpropagation process performed by the algorithm to further refine the predicted output. This process is known as training the neural network. In this research, the weights are applied to the dense layer thickness and porosity of porous section as inputs, leading to stiffness of stems, stresses in dense layer and porous sections, and factor of safety as outputs. Neural networks can be and already are being used in almost all fields of scientific studies. Figure 3 shows a basic fully connected neural network schematic where $w_{i}$ represent the weights, il is the input layer, $hl_{i}$ are the hidden layers, and ol is the output layer. 

Each of the machine learning predictors is used to fit the data 10 times according to the NumPy seed in the range of [0, 9] which creates a sampling with a replacement scheme. The algorithms are trained on the 70% training portion and validated on the 30% validation portion of the dataset. This approach creates 60 different runs that were implemented on Python 3 [38]. The validation root mean squared error (RMSE) and mean absolute percentage error (MAPE) are reported in the results and discussion section for each of the 60 runs. RMSE and MAPE are calculated using Eqs. 7 and 8, respectively.

(7) $$RMSE=\sqrt{\frac{1}{n}\overset{n}{\underset{t=1}{\sum }}{\left(A_{t}-P_{t}\right)}^{2}}$$

(8) $$MAPE=\left(\frac{1}{n}\overset{n}{\underset{t=1}{\sum }}\frac{\left|A_{t}-P_{t}\right|}{A_{t}}\right)\cdot 100\%$$

where *A* is the actual value and *P* is the predicted value. RMSE and MAPE results are reported in the results and discussion section where a comprehensive comparison is shown across the six utilized machine learning algorithms and their associated samples. An average, standard deviation, maximum, and minimum result from Equations (7) and (8) is also shown for the purposes of clearly determining the lowest validation prediction error.

Additionally, the trained algorithms are tested against an unseen test set. The test set inputs are shown in Table 3 where different design parameters are chosen to truly test the algorithms’ feasibility in accurately following the trend of the training and validation sets’ output variables.

After using the trained and validated models to predict the outputs of the test set, the trend is observed, and the best algorithm is chosen based on a voting system. The voting system utilizes the proportional trend validity for each algorithm and produces a score out of 100. The best algorithm is then used to showcase its trend results for each of the four output variables. The criteria for choosing the best algorithm depend on two factors. The first factor is the monotonicity of the prediction where no two consecutive predicted outputs are repeated. To clarify, monotonicity means always increasing or always decreasing. The second factor is the nonexistence of negative value predictions as some negative values are wrongly predicted by some algorithms where no negative values exist in the original data. 

## 3. Results and Discussion

### 3.1. Finite Element Analysis

This study investigates the biomechanical performance of semi-porous hip stems to address issues such as stress shielding, which mainly occurs in Gruen Zone 7 due to a solid dense metallic stem. Designing porous and semi-porous implants can address the complications of stiff dense implants. However, in silico, in vitro, and in vivo investigations of the newly designed porous and semi-porous complex structure are time-consuming and costly; therefore, the results of computer simulations were validated using machine learning in this study. After validation, the algorithms were trained and employed to predict the biomechanical performance of newly designed semi-porous hip stems. Finite element analysis of implants showed that by increasing the thickness of the outer layer, the stresses in the layer were decreased, as discussed in a previous study [12]. In addition, by increasing the porosity, the stresses were decreased. These thicknesses of the layer and the change in porosities affected the stiffness of the stems [12]. Similar findings were observed when a stochastic open-cell porous structure was incorporated in the porous femoral stem [39]. The results of the study were validated experimentally, and showed a reduction of 31% in flexural stiffness when a 33% porous stem was simulated compared with the dense stem. In another study [40], a cobalt-chromium implant with a porous architecture was introduced with various porosities. The results showed that the stiffness was decreased by increasing the porosity, which is in agreement with the current study. Hazlehurst et al. [41] also modeled porous and dense stems simulated in finite element analysis. The results also showed that the porous stem takes more stress as compared with the dense stem. Further validating the finite element results in [12], the new designs in this study also predicted similar trends. Increasing the layer thickness and decreasing the porosity increased the stiffness of the stem. Even while keeping the same porosity, increasing the layer thickness caused an increase in implant stiffness which was predicted by the algorithms. Similarly, keeping the same layer thickness and decreasing the porosity also caused an increase in stiffness. Moreover, increasing the thickness of the outer layer and increasing the porosity improved the factor of safety, which is also consistent with the previous study [7]. These increases in stiffness and factor of safety are good in the view of longevity of any part; however, much higher stiffness may cause stress shielding and reduce bone density around the hip stem. Therefore, an optimal design is required to satisfy both simultaneously; this will be included in future research. 

### 3.2. Machine Learning Predictions

Following the application of the machine learning methodology in Section 2.2, the results are showcased and discussed in this section. Table 4 shows the validation RMSE and MAPE results across all utilized algorithms and sample seeds. 

The lowest average MAPE in terms of percentage is the decision tree regression algorithm. However, since observing a reasonable trend is the desired outcome, DTR is excluded from being the algorithm of choice. In fact, all algorithms, after training and validation, were used to predict the test set, and the trend was observed. Table 5 shows the scoring of trend validity on the test set using the trained and validated machine learning algorithms. 

**Figure 4:**
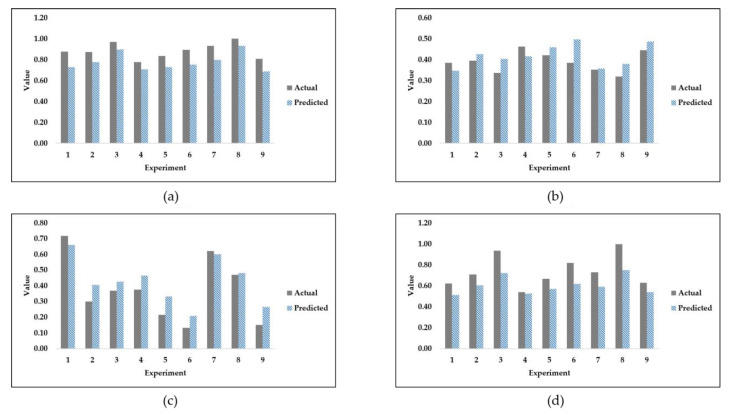
Ridge regression validation set predictions of (**a**) stiffness of stems; (**b**) maximum stresses in dense layer; (**c**) maximum stresses in porous section; and (**d**) factor of safety.

It is evident from Table 5 that the two algorithms with the best trend score are ridge and linear regression. Linear regression was excluded due to its prediction of negative values according to the second selection criterion mentioned in the methodology section. To reiterate, the criteria for choosing the best algorithm depended on two factors. The first factor is the monotonicity of the prediction, where no two consecutive predicted outputs are repeated. The second factor is the nonexistence of negative value predictions. In Table 5, a score of 0 or 1 is used to describe criteria satisfaction, where a score of 1 indicates that the criterion of monotonicity is met, whereas a score of 0 indicates that monotonicity is not met. Figure 4 shows the validation prediction of SS, MSDL, MSPS, and FS, using the ridge regression algorithm on the fourth sample seed.

**Figure 5:**
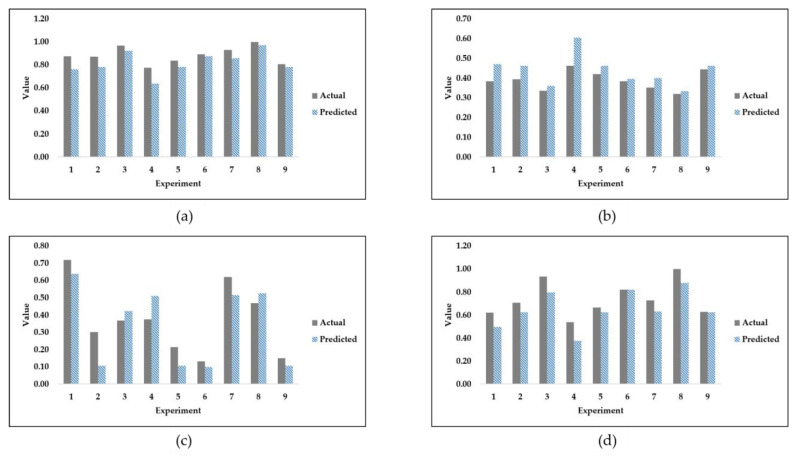
Decision tree regression validation set predictions of (**a**) stiffness of stems; (**b**) maximum stresses in dense layer; (**c**) maximum stresses in porous section; and (**d**) factor of safety.

As shown in Figure 4, ridge regression is able to reasonably predict the validation set outputs. However, ridge regression is not the best algorithm in terms of average validation accuracy. On the one hand, it is noted that the ridge regression algorithm under-predicts the values of the stiffness of stems and the factor of safety. On the other hand, the predictions of maximum stresses in the dense layer and maximum stresses in the porous section are each considered to be a mix of both over-prediction and under-prediction. For the purposes of comparison, and since the fourth sample seed was used to produce Figure 4, DTR is used to produce Figure 5 as follows.

Figure 5 is illustrated to showcase the validation prediction of DTR which is the highest-performing algorithm in terms of average validation MAPE. Nonetheless, the trend of test set predictions as a result of using DTR does not satisfy the criteria established in this paper. As shown in Figure 5, decision tree regression is able to more reasonably predict the validation set outputs as compared with ridge regression. In fact, it is the best-performing machine learning algorithm based on the average MAPE results. Decision tree regression mostly under-predicts the true values of stiffness of stems and the factor of safety. The algorithm of DTR strictly over-predicts maximum stresses in the dense layer while producing a mixture of over- and under-predictions for the maximum stresses in the porous section. 

**Figure 6:**
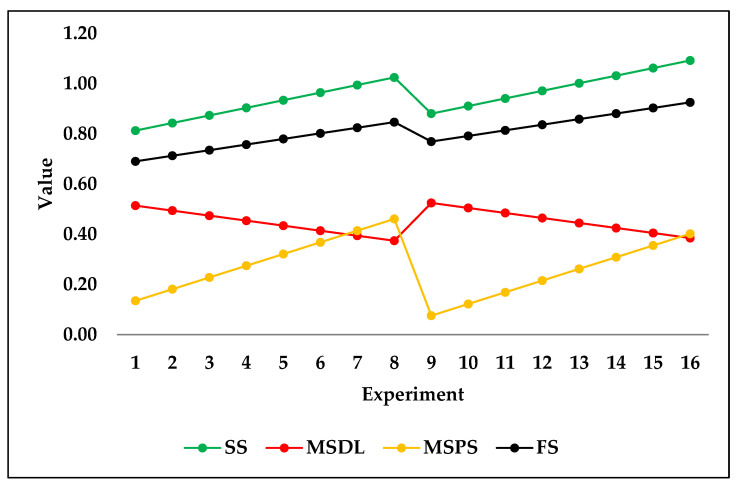
Ridge regression test set predictions of all four output variables.

The following figure, Figure 6, shows the test set trend of predicted results based on input design variables of the hip stem using the ridge algorithm as trained on the fourth sample seed. The predicted results show that the stiffness of the stem, maximum stresses in the porous section, and factor of safety were increased when porosity was decreased while keeping the same outer layer thickness of 2.5 mm. A similar trend was observed when the outer layer thickness was 3 mm and porosity was decreased. Contrarily, the stresses in porous sections were decreased by decreasing the porosity of stems in both outer layer thicknesses of 2.5 and 3 mm. The trend of these predicted results agreed with the validated results of finite element simulations. The increase in stiffness of the stem by decreasing the porosity is logical because the amount of material in the stem was increased, which resists more against loads. Similarly, the stresses in the dense layer were increased by decreasing the porosity, which showed that the dense section started taking more load as the material of the porous section was decreased. In addition, the factor of safety usually increases when the volume of material is increased, and this was the case in the prediction. However, the stresses in the porous section were decreased by decreasing the porosity because the porous material was not able to take greater loads and yielded. 

It is clear that the trends of the predicted test set, as shown in Figure 6, are logical and in agreement with the original dataset. For this reason, it can be concluded that ridge regression can be used for the purposes of future design considerations without the need for further simulation experiments. The necessity to conduct simulation experiments is time-consuming and may be completely replaced by machine learning prediction efforts when the algorithms are fully optimized and proven to perfectly perform predictions.

**Figure 7:**
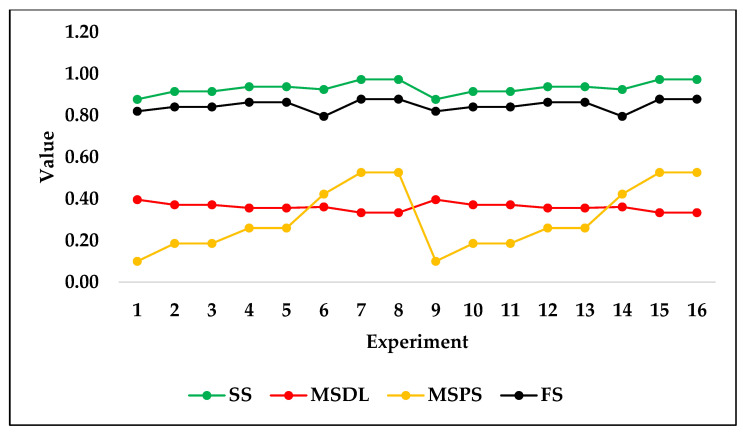
Decision tree regression test set predictions of all four output variables.

It is evident that the trend produced by the ridge regression predictions of the test set followed the original dataset’s trend in terms of the output variables *v*_3_ to *v*_6_, stiffness of stems, maximum stresses in a dense layer, maximum stresses in a porous section, and factor of safety. For the purposes of comparison, DTR was also tested on the same test set. As can be seen in Figure 7, the trend of the predicted test set using DTR is a poor representation of the original dataset’s trend in terms of the output variables *v*_3_ to *v*_6_. Figure 7 shows that almost every two successive predicted outputs have the same level, which does not show realistic results, as shown in Figure 6 for ridge regression. When the outer layer thickness or porosity is changed, the stiffness of the stem and stresses in the dense and porous sections should be changed, which is not accurately predicted by DTR. 

**Figure 8:**
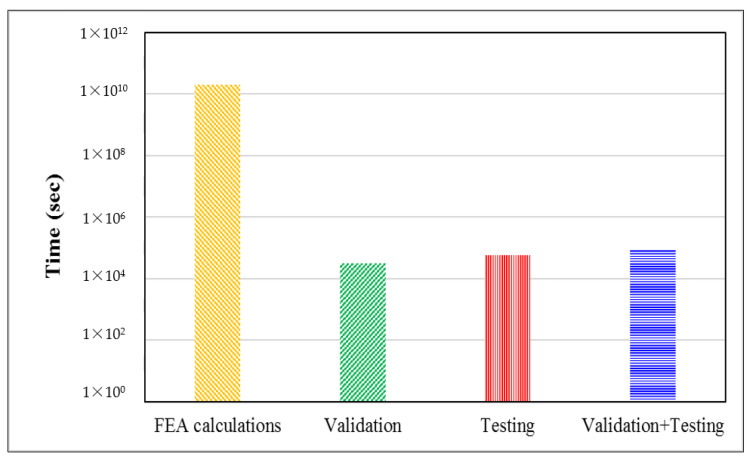
The computational time for the biomechanical performance of hip stems required by FEA calculations and ML.

Figure 8 shows the calculation times of FEA calculations, ML validation, testing, and total ML time. Typically, it takes hours to a few days to perform finite element calculations through modeling and finite element analysis, whereas machine learning models take significantly less time. Even if machine learning training time is also taken into account, the total time required is still significantly lower than that taken for FEA calculations. Hence, machine learning models are computationally more efficient and significantly accelerate the predictions of the biomechanical performance of new designs for hip stems. In addition to time reduction, it is worth noting that these results were also obtained using a relatively small data set.

The framework of this study has great potential to aid new stem designs, and thus may be applicable for clinical use. It allows for the rapid exploration of the biomechanical performance of various designs of hip stems to be used and applied to the personal conditions of patients. Thus, this study has reduced the computational cost and time for the design process of stems. In general, machine learning models show robust performance in predicting the biomechanical performance of different designs of hip stems; however, the prediction efficiency can always be improved by investigating a larger dataset. 

## 4. Conclusions

Finite element simulations are computationally expensive as compared with machine learning algorithms, but give more accurate results. The validation of finite element results was successfully achieved using machine learning algorithms and this validation of finite element results would be far too expensive in in vivo experimentations.

Using the simulated data, a variety of machine learning algorithms were utilized for the purposes of predicting the stiffness of stems, maximum stresses in a dense layer, maximum stresses in a porous section, and factor of safety. The ridge regression algorithm was shown to produce the most accurate test set prediction in terms of trend as compared with the original dataset’s trend. Thus, the trend of biomechanical performance of new stem designs was successfully predicted using trained machine learning algorithms. 

Future research potential depends on further optimizing machine learning algorithms to achieve a better prediction scheme for the biomechanical performance of different stem designs. An example of further optimization could depend on using grid search to optimize the selected hyperparameter values. Another avenue of future research could include utilizing different test sets to facilitate machine-learning-informed implant design without the need for running computer simulations. In addition, future research can also focus on using machine learning techniques in the designing of high-performing hip implants rather than simply predicting design parameters.

One of the limitations of this paper is the relatively small dataset that was used to train the machine learning models. Accordingly, the accuracy of the predictive models was affected. The use of a larger dataset may improve the accuracy of the prediction. Another limitation is that these results are predicted based on finite element analysis and machine learning approaches; however, the biomechanical performance should be investigated using in vivo experiments. Another limitation is the fact that ML model complexity was not considered when evaluating the models used in this research. More complex models are usually expected to perform better but they are less interpretable. The current algorithms are not trained to predict biochemical effects, including biocompatibility, stress shielding, and bone remodeling, which are gradual changes in the peri-stem bone changes and are important in assessing the secondary stability of implants. Hip stems can be optimized considering changes in bone density to maximize the life of total hip arthroplasty. Once the implant design is optimized, a biochemical study should be carried out to study the effect of material and design on biological response.

## Figures and Tables

**Table 1:** Dataset details.

Simulation	DLT	PPS	SS	MSDL	MSPS	FS
1	0	90	134	90	104	0.165116842
2	0	77	298	173	136	0.273558063
3	0	63	542	189	217	0.311399648
4	0	47	844	189	312	0.355395013
5	0	30	1284	189	296	0.649761978
6	0	18	1671	189	289	0.956662736
7	0.5	90	900	969	31	0.439016544
8	0.5	77	999	848	64	0.504168444
9	0.5	63	1141	709	106	0.607191898
10	0.5	47	1321	583	147	0.754651623
11	0.5	30	1593	452	190	1.012067069
12	0.5	18	1845	368	217	1.274545641
13	1	90	1340	578	15	0.870252011
14	1	77	1412	540	35	0.906265976
15	1	63	1508	493	65	0.978832319
16	1	47	1626	445	101	1.09870317
17	1	30	1803	383	148	1.299523956
18	1	18	1967	337	184	1.503057065
19	1.5	90	1639	445	10	1.277959118
20	1.5	77	1691	427	25	1.288267624
21	1.5	63	1757	403	47	1.371124031
22	1.5	47	1835	378	76	1.459330144
23	1.5	30	1949	345	117	1.644608986
24	1.5	18	2055	318	152	1.820361118
25	2	90	1845	379	8	1.696200284
26	2	77	1883	368	19	1.6979466
27	2	63	1928	355	37	1.740895669
28	2	47	1978	340	62	1.787807737
29	2	30	2050	321	99	1.943079537
30	2	18	2115	305	133	2.079133065

**Table 2:** Selected default hyperparameter values for utilized algorithms.

Algorithm	Hyperparameter	Hyperparameter Value
Decision tree regression	Criterion	Squared error
	Splitter	Best
Linear, ridge, lasso, elastic net	Alpha	1.0
	Fit intercept	True
Multilayer perceptron	Activation	Rectified linear unit (Relu)
	Hidden layer sizes	100
	Solver	Adaptive momentum (Adam)

**Table 3:** Test set values.

#	DLT	PPS
1	2.5	80
2	2.5	70
3	2.5	60
4	2.5	50
5	2.5	40
6	2.5	30
7	2.5	20
8	2.5	10
9	3	80
10	3	70
11	3	60
12	3	50
13	3	40
14	3	30
15	3	20
16	3	10

**Table 4:** RMSE and MAPE comparison.

	DTR		LR		RR		LSR		EN		MLP	
Seed	RMSE	MAPE	RMSE	MAPE	RMSE	MAPE	RMSE	MAPE	RMSE	MAPE	RMSE	MAPE
0	0.11	16.91	0.10	24.22	0.13	25.88	0.22	49.70	0.22	49.70	0.14	32.13
1	0.08	13.87	0.10	20.63	0.13	26.59	0.21	47.98	0.21	47.98	0.14	30.28
2	0.11	28.10	0.17	47.35	0.22	76.06	0.30	106.34	0.30	106.34	0.21	70.74
3	0.10	16.99	0.08	18.72	0.12	27.44	0.20	46.70	0.20	46.70	0.10	20.67
4	0.09	15.29	0.07	12.54	0.11	18.21	0.22	37.53	0.22	37.53	0.07	13.39
5	0.11	16.60	0.09	21.73	0.13	27.34	0.23	52.72	0.23	52.72	0.11	25.69
6	0.10	22.75	0.15	31.14	0.19	52.62	0.27	79.88	0.27	79.88	0.19	52.65
7	0.09	17.10	0.13	35.67	0.16	42.26	0.25	72.02	0.25	72.02	0.21	55.02
8	0.11	26.50	0.15	48.14	0.19	67.98	0.28	99.77	0.28	99.77	0.19	64.38
9	0.15	22.04	0.14	20.11	0.19	34.35	0.27	53.36	0.27	53.36	0.19	30.10
Average	0.10	19.62	0.12	28.03	0.16	39.87	0.24	64.60	0.24	64.60	0.16	39.50
Sample σ	0.02	4.90	0.03	12.22	0.04	19.58	0.03	23.77	0.03	23.77	0.05	19.62
Max	0.15	28.10	0.17	48.14	0.22	76.06	0.30	106.34	0.30	106.34	0.21	70.74
Min	0.08	13.87	0.07	12.54	0.11	18.21	0.20	37.53	0.20	37.53	0.07	13.39

**Table 5:** Trend scores based on the test set.

	Trend Scores					
Seed	DTR	LR	RR	LSR	EN	MLP
0	0	1	1	0	0	1
1	0	1	1	0	0	1
2	0	1	1	0	0	0
3	0	1	1	0	0	1
4	0	1	1	0	0	1
5	0	1	1	0	0	1
6	0	1	1	0	0	0
7	0	1	1	0	0	1
8	0	1	1	0	0	0
9	0	1	1	0	0	0
Trend Score	0	10	10	0	0	6

## Data Availability

Data is available in Table 1 and Table 3.
